# Preface to ‘Advanced neurotechnologies: translating innovation for health and well-being’

**DOI:** 10.1098/rsta.2021.0004

**Published:** 2022-07-25

**Authors:** Rupam Das, Giulia Curia, Hadi Heidari

**Affiliations:** ^1^ James Watt School of Engineering, University of Glasgow, Glasgow G12 8QQ, UK; ^2^ Department of Biomedical, Metabolic and Neural Sciences, University of Modena and Reggio Emilia, Modena, Emilia-Romagna, Italy

**Keywords:** neurotechnology, biomedical engineering, neural engineering

In the nervous system, huge amounts of neurons constantly induce and transmit electrophysiological signals to communicate between neurons and brain regions. Innovative neurotechnologies have added significantly to neural engineering and have led directly to many fundamental scientific insights into the function of the central and peripheral nervous systems. This issue addressed the novel approaches toward biocompatible neural interfaces, thin and flexible electronics and wireless circuits and systems, which will allow readers to identify the requirements, challenges and future directions related to biointegrated implantable neurotechnologies. Additionally, this special issue aims to report the latest advances and future trends of critical techniques and frameworks in implantable neural devices, which will allow biomedical researchers to identify new opportunities.

The theme issue starts with a research article by Lee & Fried [1], which aims to show that innovative magnetic stimulation of the visual cortex (V1) using microcoils induces spatially confined activation in the secondary visual cortex (V2) in mouse brain slices. They demonstrated that, compared with the traditional electrical stimulation, the microcoils-based magnetic stimulation is better for confining the activation to a small region in V1 and produces more precise and sustained activation in V2. The finding that microcoil-based stimulation propagates to higher visual centres raises the possibility that complex visual perception, e.g. that requires sustained synaptic inputs, may be achievable.

In a thought-provoking article by Luca Selmi and colleagues [2], after discussing recent progress in electronic sensor architecture for *in vitro* neural recording, authors modelled the neuron/sensor interface and readout electronics with a mixed-mode finite-element/circuit approach. Their proposed simulation model acts as a bridge between hardware and wetware, thus enabling simultaneous optimization of the readout and the sensor device by taking into consideration of neuron environment.

In an interesting article [3] by Kim & Ho reviews the recent advances in the development of wireless interfaces for brain neurotechnologies. They summarize the state-of-the-art requirements for brain-implanted devices to realize the wireless interface and address the operating principles and applications of wireless interfaces based on modalities. In addition to discussing challenges related to wireless brain neurotechnologies, this article examines emerging solutions allowed by current developments in electrical engineering and materials science.

Appropriate material selection and careful design are the most crucial considerations to ensure successful, chronic neural probe implantation. The subsequent research article [4] by Eve McGlynn *et al.* employs a round of simulation to investigate the robustness of the probes concerning the forces induced during surgical implantation. They also numerically and experimentally identified the optimum probe design from a pool of four, based on buckling force, insertion success rate, three-point bending tests and a magnetic resonance imaging compatibility test.

Polymer-based flexible neural probes offer superior biocompatibility due to enhanced surface biochemistry and mechanical compliancy compared with the traditional rigid (Si or metal) probes. Regardless of the push toward flexible consumer electronics, there are few studies that aim to bring the fabrication of electronics on flexible polymer substrates to the nano-regime. The article by Finlay Walton *et al.* [5] explores the challenges during fabrication by using photolithography and complementary techniques in a cleanroom to develop flexible, nano-scale neural probes.

In the following article, Kianoush Nazarpour and colleagues investigated how informative features can be extracted from population activity electroneurographic (ENG) signals [6]. To do this, they used several feature extraction frameworks on sensory ENG datasets, and their classification performance was compared. Their proposed feature extraction framework, which incorporates spatio-temporal focus and dynamic time warping, achieved classification accuracies greater than 90% while keeping a low computational cost. Thus, this research has extended the tools available to extract features from sensory population activity ENG signals.

Upper-limb prostheses research is typically limited to laboratory-based due to inefficient communication loops between users, manufacturers and clinicians. In an exciting article Kianoush Nazarpour *et al.* demonstrated and proposed an alternative paradigm by using the Internet of Things (IoT) setting, which allows remote data collection, real-time visualization and prosthesis reprogramming through Wi-Fi and a commercial cloud portal to show the feasibility of beyond-the-laboratory prosthesis research according to user needs [7].

In the following article, Javad Ahmadi-Farsani *et al.* introduces a hybrid memristive system in which the experimental operation of a spiking neural network circuit using a 4 × 4 1T1R synaptic crossbar together with four post-synaptic CMOS circuits is demonstrated [8]. It also illustrates one-shot winner-takes-all training and stochastic-binary spike-timing-dependent-plasticity learning using their proposed system.

Ángel Canal-Alonso *et al.* developed a miniaturized vagus nerve stimulator (VNS) for application in epilepsy research for laboratory animals as the available VNS devices for human are not only expensive for animal research but also too large [9]. The proposed device reduces the expenditure and was validated in hamsters.

Tactile feedback is critical in a wide range of human–machine interfacing (e.g. virtual reality, teleoperation and prosthetics). However, currently available tactile feedback interfaces consist of few stimulation and sensing units, thus limiting the extent of data/information transmitted to the user. The research article by Yahya Abbass *et al*. introduces a new technology that comprises arrays of flexible sensors integrated into the fingers and palm of a robotic hand, embedded electronics, a multichannel stimulator and flexible electrodes placed on the subject's hand to convey comprehensive tactile feedback to the user of a robotic end-effector [10]. Furthermore, they experimentally demonstrated that distributed sensing resulted in substantially better performance with satisfactory accuracy for both static and dynamic patterns. The proposed system could be a significant step in the direction of the advancement of a high-density human–machine interfacing.

Brain neurotransmitter sensing is vital for the understanding of neurodegenerative diseases. The article by Mounir Boukadoum and colleagues describes and evaluates the design of a novel, compact and non-invasive instrument for neurotransmitter detection based on the colorimetric sensing method compared with the currently available bulky instruments. The proposed instrument is tested with gold nanoparticles, and its performance is compared with that of a commercial instrument, showing that the designed prototype matches the commercial instrument in performance while being physically much smaller, and it can surpass it with further improvements [11].

We would like to thank all the authors wholeheartedly for their invaluable contributions in shaping this issue with a collection of papers on advanced neural technologies, a topic of significant importance to enable our understanding of the complex human brain and identify challenges toward developing the next generation of neural interfaces.

## Data Availability

This article has no additional data.
